# Open-label placebo treatment for reducing overeating in children: A study protocol for a randomized clinical trial with an app-assisted approach

**DOI:** 10.1016/j.conctc.2023.101175

**Published:** 2023-06-20

**Authors:** Anne Schienle, Isabella Unger

**Affiliations:** Clinical Psychology, University of Graz, BioTechMed Graz, Austria

**Keywords:** Open-label placebo, Craving, Binge-eating, Children, App-assisted

## Abstract

Food advertising has become almost ubiquitous in Western societies. In adults as well as in children this omnipresence of food cues has been shown to trigger cravings and overeating, which can lead to overweight or even obesity. This is concerning because obesity is a leading cause of preventable diseases. The planned project aims at reducing craving and overeating in overweight/obese children using a placebo treatment. A total of 80 children (40 girls, 40 boys; aged between 8 and 12 years; body mass index >90th percentile) will take part in the study. A randomized controlled cross-over design will be implemented, which will include four weeks with daily placebo treatment and four weeks without placebo treatment. The placebo will be introduced without deception as an open-label placebo (OLP), that can help to control food cravings. The study will use an app-assisted approach: The children will rate the intensity of their cravings, the occurrence of binge-eating episodes, their emotional state, and placebo usage via a smartphone application. It is expected that the OLP will help the children to reduce cravings and body weight. If effective, this OLP approach could be implemented in weight-control programs for children.

## 1 Introduction

The prevalence of overweight and obesity has severely increased during the past decades. According to the [1]; worldwide obesity has nearly tripled since 1975; this also includes a dramatic increase in childhood obesity. In Austria, the Childhood Obesity Surveillance Initiative of the [1] has indicated that 30% of all boys and 25% of all girls aged between eight and nine years are overweight or obese.

Obesity in childhood is particularly concerning due to its negative influence on children's physical health and psychosocial development. Being overweight in childhood is associated with greater risk and earlier onset of chronic disease (e.g., type 2 diabetes, cardiovascular disease), adverse psychosocial consequences (e.g., low self-esteem, depression, peer rejection), and lower educational attainment (for a summary see Ref. [2]).

Various forms of overeating, such as binge eating (episodic overeating with loss of control) contribute to the development of overweight/obesity [3]. Moreover, binge eating is associated with a further increase in body mass index (and body fat mass) in children who are already overweight [4]. Thus, interventions concerning such challenging forms of overeating may be helpful to counteract negative health outcomes.

Overeating (binge eating) can be triggered by food cues (signals in the environment that indicate the presence of food; see meta-analysis by Ref. [5]. In Western societies, people are constantly exposed to visual food cues, both in the virtual world (e.g., cookery shows on TV and food blogs) and in the real world (e.g., in supermarkets, and restaurants). This type of stimulation elicits appetite and the urge to consume the displayed food items (craving). Since these food cues are so prevalent, it is not surprising that individual reactivity to food cues plays such an important role; indeed, it has been shown to predict the frequency and intensity of craving, overeating, and subsequent weight gain [5,6]. A study by Ref. [7] found that in overweight children, food consumption was triggered more often by visual food cues, compared to normal-weight children. Thus, overweight children appear to be more vulnerable to overeating when presented with food cues. This further underscores the importance of interventions for this group which aim at reducing overeating.

Unfortunately, eating/weight control programs (diets, exercise programs) often only produce positive short-term effects [2]. Further, medications are available for reducing appetite and excessive weight gain. However, these appetite suppressants show undesirable side effects, such as gastrointestinal irritation, cardiovascular problems, and even an increase in symptoms of mood-related disorders [8]. Thus, these medications cannot be considered the best treatment option for overweight, especially in children. Therefore, due to the lack of successful and low-risk treatment options available, the current study will investigate a further intervention, and test the effects of a placebo for reducing appetite and episodic overeating.

It has been demonstrated previously that placebos can positively influence appetite regulation. This has been shown, for instance, in placebo-controlled clinical trials that have investigated appetite suppressants, as well as in placebo studies with healthy adults and patients diagnosed with binge-eating disorder (for a summary of the literature see Ref. [9]. However, it should be noted that these positive changes were achieved through deceptive placebo treatment. This means that the placebo recipients were not informed about the true nature of the treatment, which violates the ethical principles of respect for the autonomy of patients. Importantly, the ethical issues surrounding deceptive placebo treatment can be circumvented by the use of open-label placebos (OLPs). With OLP treatment, placebo recipients are fully informed that they receive a substance or intervention that is not directly known to cause an effect on a certain outcome [10]. The clinical efficacy of OLPs compared with no treatment was investigated by Ref. [11] in a meta-analysis (5 studies; n = 260 adult participants). The disorders which were treated in those studies included irritable bowel syndrome (IBS), depression, allergic rhinitis, back pain, and attention deficit hyperactivity disorder (ADHD). Overall, OLP treatment was found to cause significant symptom reduction, suggesting that OLPs can improve various clinical conditions. To date, only three studies have tested OLP treatment in children and adolescents [[12], [13], [14]]. The authors of those studies reported significant symptom reduction in patients with ADHD [12,13] and functional abdominal pain or IBS [14]. The majority of the children found the placebo to be useful [13] and no adverse treatment effects occurred [14].

Considering these promising results, as well as the fact that placebo response rates in clinical trials with children are higher than the response rates of adults [15], this clinical trial will test OLP effects on reducing overeating in overweight/obese children. It is planned that the placebo treatment will be continued daily over four weeks. Placebo administration will be supported by a smartphone application that helps to monitor cravings, binge eating, the affective state and placebo intake of the children. To optimize treatment effects, participants will be instructed to take the placebo daily in the morning and additionally on an as-needed basis (specifically, when they have been exposed to food cues and feel that their urge to eat has increased substantially).

Additionally, variables (e.g., self-esteem, depression, lack of physical activity) that have been associated with overeating will be assessed (e.g., Refs. [[16], [17], [18]]. It will be investigated whether these variables are associated with the placebo effect.

## 2 Methods

### 2.1 Sample

The planned sample consists of 80 children (40 overweight/obese girls, 40 overweight/obese boys). A power analysis calculated with G*Power (version: 3.1.9.2.; [19] showed that for a medium effect size of f = .25, with an alpha level of 0.05 and 95% power, the required sample size is 72.

Inclusion criteria are age between 8 and 12 years, BMI >90th percentile (relative to the age/gender group; [20], and ownership of a smartphone. Exclusion criteria are medication (e.g., antidepressants, appetite suppressants), reported diagnoses of somatic diseases (e.g., neurological disorders, digestive problems), mental disorders (e.g., ADHD, eating disorders), and puberty (e.g., as indexed by menstruation, voice fluctuation).

### 2.2 Design

The study has a randomized, controlled cross-over design with two conditions (OLP vs. no OLP). The participants will be randomly allocated (random number table) to one of two sequences of treatment: four weeks with OLP treatment followed by four weeks without OLP treatment, or four weeks without OLP treatment followed by four weeks with OLP treatment. By using this crossover design, each participant serves as his/her own matched control. This increases statistical power. In between the two conditions, there will be a four-week break to prevent carry-over effects.

### 2.3 Open-label placebo

Distilled water with blue food coloring (ye E133 brilliant blue FCF) will be presented as a manual pump spray in a 30 ml glass bottle. The placebo is administered orally in the morning and can be taken as needed when food cravings increase substantially.

The parents receive information about placebos that follow the instruction described in the study by [30]. It will be stated that placebos made of an inert substance (like sugar pills) have been shown in scientific studies to produce significant improvement in various conditions. The participants are further informed that the placebo effect is powerful, the body can learn to respond to placebos through conditioning, positive expectations/beliefs are helpful but not necessary, and that taking the placebo faithfully is crucial [30]. A child version of this information sheet (instruction leaflet) will be provided to the participating children.

### 2.4 Smartphone app

The child provides information concerning his/her food cravings, the occurrence of binge eating episodes, placebo dosage (number of incidents when the placebo was used), and mood via a smartphone app that has been created for this project. It was our goal to develop a 'kid-friendly' app with an emphasis on a visually appealing and easy-to-navigate interface. The data gathering is achieved by combining a Progressive Web App and a remote server for storage. A unique code is assigned to each participant and then encrypted and saved through an SSL-certified website. The survey is a webpage created through the use of HTML, CSS, and Javascript (using the Vue.js Framework). After completing the survey, the data are sent to a remote server, where a Python Flask script handles the data collection and creates a CSV file participants code containing all the important information.

Each child will be introduced individually to the handling of the app during the first diagnostic session. We will install and access the app together with the child and go through each of the questions. The child is reminded via a pop-up function to open the app every evening, before bedtime. The average mood during the day will be evaluated via slider ratings anchored by a frowny/smiley (from very negative to very positive). Then, the child is asked: ‘How strong were your cravings today?’ (rating via a scale from 0 to 100 (‘not strong’ - ‘very strong)’, and ‘How many eating attacks did you have today?’ indicating a number). Additionally, the child is asked how often he/she has taken the OLP ‘How often did you use the placebo today?’

At the beginning of the study, the child rates the expected efficacy of the placebo (1–100). At the end of the study, the perceived OLP effectiveness is evaluated (‘How much did the placebo reduce your cravings?” answering mode 1–100: ‘not at all’ - ‘very much’). Moreover, children will be asked to rate how difficult it was for them to use the app (0–100; ‘not difficult at all’ – ‘very difficult’).

The app offers the possibility to read the information (as described in the consent form, on the information sheet, and the instruction leaflet) about the placebo again and has an additional window for comments.

### 2.5 Procedure

This study (which is currently in the enrolling by invitation phase) follows the recommendations of the declaration of the World Medical Association of Helsinki (revised version, 2000) and the Good Clinical Practice (GCP) – Guidelines (CPMP/ICH/135/95, Final Approval by CPMP 17/07/96). The project has been approved by the ethics committee of the University of Graz (GZ. 39/26/63 ex 2019/20) and has been registered as a clinical trial on the German Register for Clinical Studies (DRKS00029251, 06/07/2022). The project is funded by the Austrian Science Fund (FWF: KLI 1062-B).

Children will be recruited via schools and social media. After a short telephone interview with the parents (to check for inclusion and exclusion criteria), appointments will be scheduled to give the children and their caregivers detailed information about the study. Furthermore, informed consent will be obtained from each participant and the parent/caregiver.

In the placebo condition, the participants receive the placebo and are informed about the treatment. After four weeks of placebo usage, the children are asked to return the bottle to measure placebo intake (in ml). In the control condition, the participants are instructed to continue with their usual eating behavior.

**Fig. 1:**
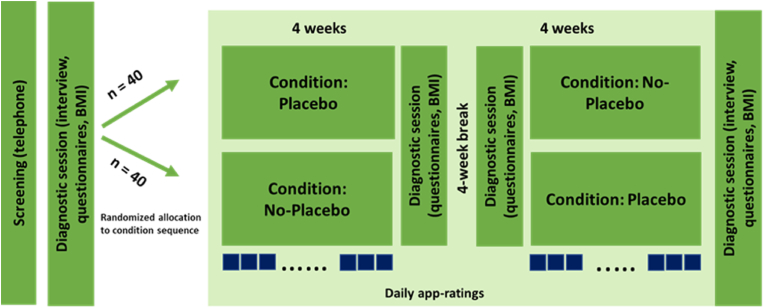
Procedure.

In the first diagnostic session, the app is installed and explained. Moreover, each child receives an activity tracker that assesses the distance walked per day. The tracker has no display so that the children are not distracted by information provided by an additional device (besides the smartphone). Physical activity is assessed as a control variable. Finally, questionnaires are answered at the beginning of the study, at the end of the first condition, after the break, and at the end of the second condition. Moreover, the body weight/height is measured at each time point (the procedure is depicted in Fig. 1).

In the first diagnostic session (in person at the university), parents will complete the Child Behavior Checklist (CBCL/4–18; parents' version (Working Group German Child Behavior Checklist, in German, [21]. The questionnaire assesses parents' judgment of social competencies as well as behavioral and emotional problems of their children. The items are rated on a 3-point scale ranging from 0 = ‘not applicable’ to 2 = ‘accurate/often applicable’. Retest reliability after five weeks is Cronbach's alpha = .81. Moreover, the adapted version of the Home Environment Interview (HEI, [22], will be conducted with the caregivers. The adapted HEI examines fruit and vegetable access at home, food consumption of the child, fast food consumption, the consumption of sweetened non-alcoholic beverages, mealtimes together as a family, and physical activity of the child and the caregivers. Additionally, it covers questions about criteria for puberty such as menstruation, pubic hair, breast growth and acne for girls and voice fluctuations, pubic hair, and acne for boys. Fianlly, the child's weight development (throughout childhood: baby, toddler) is covered.

Children will complete the following questionnaires during the diagnostic sessions at the university:

• Satisfaction With Life Scale for Children (SWLS-C, [23]. The scale consists of five items (e.g., ‘In most respects, my life is as I want it to be.’), measured on a 5-point Likert scale. Retest reliability is r = .80.
• Depression Test for Children (DTK, [24]. The DTK measures ‘self-esteem problems/dysphoric mood’, ‘agitated behavior’, and ‘fatigue and other psychosomatic aspects’. It consists of 55 questions with a dichotomous (yes/no) response format. Retest reliability (r) ranges between 0.74 and 0.88.
• The Self-Control Scale (German version SCS-K-D Selbstkontroll-Kapazität Skala, [25]; brief version of SCS Self-Control Scale, [26], measures interindividual differences in perceived self-control capacity (retest reliability: r = .82). The scale includes items such as ‘I am good at resisting a temptation”. The brief version consists of 13 items and is measured on a 5-point Likert scale ranging from completely inaccurate (1) to applies exactly (5).
• Self-Assessment of Pubertal Maturation [27]: a gender-specific self-assessment questionnaire for children with illustrations of the 5 pubertal stages including an explanatory text. It examines the first signs of puberty for male genitalia, testicular size, male pubic hair, female breasts, and female pubic hair.
• ‘Typical day in my life’: The questions cover the children's food consumption and eating patterns of a typical day. Example questions are: ‘What do you typically eat for breakfast?” or ‘Is there a specific time when you have dinner?”. It also covers snacking routines.

In addition, the Diagnostic Interview for Mental Disorders in Children [28] will be used by a clinical psychologist to screen for possible comorbid mental disorders and exclusion criteria.

At the end of the intervention, a qualitative interview will be conducted separately with children and parents. This interview aims at identifying the subjective theory of the children/parents on how the OLP worked (placebo mechanisms). Following the suggestions by Ref. [29]; we will additionally ask whether characteristics of the treatment context influenced the perceived effectiveness of the intervention (external context: e.g., appearance/user-friendliness of the app; the behavior of others during the intervention/social support), internal context: e.g., emotions, and treatment cues: e.g., the blue placebo).

### 2.6 Hypotheses and data analysis

a) During the OLP condition (compared to the control condition), the participants will report lower levels of food cravings and a reduced number of binge episodes.
b) After four weeks of OLP treatment, the body weight will be reduced.

It will also be investigated whether the affective ratings (mood) and the activity level of the participants differ between the two conditions (OLP vs. no OLP). Statistical analyses will be carried out by using linear mixed models which are more flexible in terms of longitudinal designs relative to repeated-measures analyses of variance. For example, the dependent variables (e.g., the average level of food cravings) will be compared between the conditions for week 1, week 2, week 3, and week 4.

Moreover, exploratory regression analyses will be used to investigate associations between the assessed variables (e.g., frequency of placebo intake, BMI, age, sex, physical activity) and the placebo effect (e.g., reduction of cravings and body weight).

The qualitative data (interview on placebo mechanisms) will be analyzed via content analysis.

## 3 Discussion

Dysfunctional eating and overweight/obesity in childhood are associated with negative effects on somatic and mental health [2]. Therefore treatment strategies to reduce overeating are urgently needed. Placebo treatment can be considered an approach without negative side effects that might help to strengthen the experience of control and to reduce feelings of loss of control over eating. The administration of OLPs circumvents ethical issues associated with traditional (deceptive) placebos [10] and has the potential to serve as an easy-to-use adjunctive treatment of existing interventions for the reduction of appetite and overeating.

The planned study will be the first investigation to examine the effects of repeated OLP treatment over a longer period of time (four weeks) in children with dysfunctional eating patterns. The project includes both subjective outcome measures (e.g., self-reported cravings, mood) and a more objective outcome measure (body weight). The data will offer insights into the effectiveness of OLPs on children's food cravings and binge eating. Additionally, the temporal course of placebo effects can be monitored daily over four weeks. Another noteworthy aspect is that the planned study will compare OLP with no treatment. Previous OLP studies in children and adolescents have used OLPs only in combination with medication [12,13]: stimulants; [14]: antispasmodic medication).

Finally, the analysis of the assessed qualitative data in the interview and questionnaire data can help to better understand the mechanism of how OLPs exert their effects. If effective, this OLP approach could be implemented in weight-control programs for children.

## Declaration of competing interest

The authors declare that they have no known competing financial interests or personal relationships that could have appeared to influence the work reported in this paper.
